# Willingness to HPV self-sampling for cervical cancer screening and its predictors among women attending outpatient clinics in Meru District, Arusha Region, Northern Tanzania

**DOI:** 10.4314/ahs.v22i2.12

**Published:** 2022-06

**Authors:** Olola Oneko, Michael J Mahande, Caroline Amour, Meridith Pollie, Cheyenne Smith, Innocent B Mboya, Madelon Finkel

**Affiliations:** 1 Department of Obstetrics and Gynaecology, Kilimanjaro Christian Medical Center, P.O. Box 3010, Moshi-Tanzania; 2 Department of Epidemiology and Biostatistics, Institute of Public Health, Kilimanjaro Christian Medical College, P.O. Box 2240, Moshi-Tanzania; 3 Weill Cornell Medicine, Department of Population Health Sciences, 402 East 67^th^ Street, New York, NY 10065; 4 School of Mathematics, Statistics & Computer Science, University of Kwazulu-Natal, Pietermaritzburg, Private Bag X01, Scottsville 3209, South Africa

**Keywords:** Cervical cancer, HPV testing, HPV self-collection, Tanzania

## Abstract

**Background:**

The ability for women to self-collect human papillomavirus (HPV) samples can potentially reduce the risk of cervical cancer and increase screening coverage.

**Objectives:**

To assess the willingness to HPV self-sampling for cervical cancer screening and its predictors among women attending outpatient clinics in Arusha region, northern Tanzania.

**Methods:**

A hospital-based cross-sectional study was conducted among 706 women aged 18–55 years in Meru District Hospital and Usa River Health Centre from March to April 2019. Face-to-face intervies were conducted using a questionnaire. Data analysis was performed using Stata version 14.0. The log-binomial regression was used to determine factors associated with willingness to self-collection of HPV samples.

**Results:**

Majority (70%) of the women were willing to self-collection of HPV samples for cervical cancer screening and was associated with attending Meru District hospital (PR=2.02, 95%CI 1.77–2.31); good knowledge about cervical cancer warning signs (PR=1.11, 95%CI 1.01–1.22), prevention (PR=1.13, 95%CI 1.04–1.20), and symptoms (PR=1.61, 95%CI 1.33–1.93); and having formal employment (PR=1.22, 95%CI 1.07–1.37).

**Conclusion:**

The majority of women were willing to self-collect HPV samples for cervical cancer screening. Self-collection is, therefore, an acceptable and viable means of screening for cervical cancer, which has great implications for Tanzania from a health policy perspective.

## Introduction

Cervical cancer is a leading cause of cancer death among women worldwide, with an incidence of over 500,000 cases and approximately 250,000 deaths each year^1^–^4^. More women die from cervical cancer than from pregnancy-related complications^2^. Paradoxically, cervical cancer is one of the most preventable and treatable forms of cancer^2^, ^3^. The global incidence of cervical cancer is estimated to be 13.1 cases per 100,000 women, but remains as high as 40.1 per 100,000 in East Africa^1^. Furthermore, mortality due to cervical cancer is also higher in developing countries, with rates in East Africa approaching 30 cases versus 6.9 cases per 100,000 worldwide^1^, ^3^. As a result, nearly 90% of cervical cancer deaths occur in the Low- and Middle-Income Countries (LMICs), with the highest-burden carried by Eastern Africa, including Tanzania^1^–^3^, ^5^.

The number of cervical cancer-related deaths has decreased substantially in developed countries since the introduction of the Pap smear screening test and HPV vaccine^5^. But this is not the case in LMICs. Numerous barriers to implementing cytology-based testing have been reported in LMICs including lack of resources, unavailability of cytology laboratories, paucity of trained providers and lack of knowledge of the benefits of screening among the local population^6^, ^7^. Furthermore, some programs have reported weeks-long delays in obtaining screening results, which lead to frequent loss to follow up^7^, ^8^. Screening using visual inspection with acetic acid (VIA), however, is a comparatively sensitive and specific screening modality to use in LMICs. VIA is an inexpensive, low-tech means for cervical cancer screening and has the added benefit of providing immediate results, unlike the Pap smear test^8^, ^9^.

Majority of cervical cancer cases are caused by the Human Papillomavirus (HPV)^3^, ^10^. Approximately 70% are caused by HPV subtypes 16 and 18^4^, ^11^. Other risk factors for cervical cancer include compromised immune status, coinfection with other sexually transmitted agents, multiparity, young age at first birth, and tobacco smoking^4^, ^5^, ^11^. LMICs have a higher prevalence of HIV/AIDS where lower average maternal age, and increased parity collectively increase the risk for cervical cancer^12^.

A decade ago, Sankaranarayanan et al., showed that screening women with a single round of HPV DNA testing was associated with a significant reduction in the number of advanced cervical cancers and deaths from cervical cancer^9^. Since that time, numerous studies have affirmed the value of HPV testing, and, in 2013, WHO recommended that LMICs use a strategy of screening with an HPV test followed by VIA and appropriate treatment^13^. Today, HPV testing is a key tool for cervical cancer prevention and detection. The two most popular HPV DNA tests include the Hybrid Capture IITM™ (Qiagen) and the CareHPVTM™ (Qiagen). Both tests are sufficiently sensitive (>90%), specific (about 84%), well-accepted, and easy to administer. The high specificity limits the likelihood of false-negative test outcomes and provides greater confidence in recommending less frequent screening compared to other screening methods such as VIA.

Typically, HPV DNA screening has been done by providers at local clinics, requiring women to travel to health facilities to have their cervical samples collected. A woman must then return to obtain the results of the test. The ability for women to self-collect specimens (i.e., where the woman takes her cervical swab in the comfort and privacy of her home) can help reduce the number of health facility visits required and hence increase screening coverage rates^14^, ^15^. Also, by eliminating the need for a pelvic examination by a trained provider, self-sample collection overcomes barriers to clinician-performed screening such as transportation and cultural or religious beliefs regarding the pelvic examination^14^, ^16^. Studies have shown that women's attitudes and acceptance of self-collected testing is high^17^–^19^.

The situation in Tanzania is serious, as cervical cancer remains the most common female cancer in this country as well as in other East African countries. Only Malawi has higher cervical cancer rates than Tanzania^10^. Although an HPV vaccination program was introduced in Tanzania in 2018^5^, screening remains limited and the vaccine prevents only 70% of cervical cancers. The HPV vaccination does not protect the several million women who are already infected^15^. By the year 2013, only 12 of the 21 regions in Tanzania had a screening centre^20^. In the Lake Region, only 14.3% of the women have been screened^21^. HPV self-collection has the potential to be a game-changer in cervical cancer screening in countries such as Tanzania. There is evidence to show that this is so^14^–^16^, ^19^, ^22^, ^23^.

The feasibility and willingness to HPV self-collect have not been fully explored in sub Saharan Africa. Assessing attitudes and knowledge about HPV self-collection and its predictors would help to design strategic interventions to increase coverage and uptake of cervical cancer screening. This study assesses the willingness and predictors of women in Meru District in Northern Tanzania to self-collect HPV samples.

## Methods

### Study Design and setting

We conducted a facility-based cross-sectional study involving women attending outpatient clinics in Meru district health facilities in Arusha region, in Northern Tanzania. The study was conducted for three weeks from mid of March to late April 2019. Meru district is one of the six districts in the Arusha region. In the year 2017, Meru District Council had a total population of 306,352, of which 156,384 (51%) were females^24^.This district was selected because it has both urban and rural components. The significant economic activities include agriculture, business, and tourism activities. The district has two hospitals, eight health facilities, and 36 dispensaries. The study was conducted in Meru District Hospital and Usa River Health Centre.

### Study population, sample size, and sampling procedure

The study included women aged 18–55 years who were receiving care in outpatient clinics of selected facilities in Meru District. The sample size was determined using the formula for estimating a single population proportion using a standard normal value under 95% confidence interval (1.96), a precision of 4%, and assuming screening practice of cervical cancer of 50%. The calculated sample size was 384 by adding a 20% non-response rate and incomplete data, the final sample size became 461 women. Women who walked into the outpatient clinics were invited, provided they met the eligibility age criteria. After receiving care in respective outpatient departments, women were approached, explained the purpose of the study, and screened for eligibility. Those who provided informed consent were recruited systematically until the minimum required sample size was achieved.

### Data collection methods and tools

Before initiating the survey, we conducted several community awareness campaigns, including engaging the district leaders to help publicize the importance of screening. The survey questionnaire was pre-tested on 10–15 women from two dispensaries and one health center (apart from those included in this study) for comprehension and clarity. Data were collected using a pre-tested, structured questionnaire adapted from a previous study, which we conducted around the Lake Zone in Tanzania^21^. This tool was designed to assess the knowledge and awareness of cervical cancer, and contained questions on socio-demographic characteristics, knowledge about cervical cancer signs, risk factors, and prevention, knowledge about the HPV vaccine, screening practices as well as favorable means of receiving cervical cancer information. Trained health care staff (e.g., nurses and medical doctors) collected the data and conducted the interviews in the private and quiet rooms within respective outpatient departments.

### Ethical considerations

The Kilimanjaro Christian Medical University College Research and Ethics Review Committee approved the study, with ethical approval number 2069. Permission to conduct the study was also obtained from the regional administrative authority of the Arusha region. Informed consent was obtained from the study participants, clearly stating potential harms and benefits of participating in the study and seeking their voluntary participation by a research nurse/research Principal investigator. Oral informed consent was obtained from each participant. Both confidentiality and privacy were adhered during the study period. De-identified personal identification numbers were used instead of names.

### Study variables

Willingness to HPV self-sampling was the main outcome of interest in this study. Participants were asked the question: “If there could be a self-sample collection method for cervical cancer, will you agree to do self-sample collection (vaginal swabs) for HPV testing?” (With Yes/No response). The independent variables included respondent socio-demographic characteristics (i.e., name of health facility, age in whole years, marital status, education level occupation, having health insurance or not, and parity). We also included variables on cervical cancer knowledge of risk factors, signs of cervical cancer, and prevention. We assessed the knowledge of signs and risk factors for cervical cancer using 11-item questionnaire; for prevention we used a 5-item questionnaire. Following previous literature, we scored each correct response as 1, with incorrect or “do not know” answers scored as 0. We used a cut-off point of 50% correction rate to categorize respondents into two groups. The total score ranging from 6 to 11 or 3 to 5 were defined as a good level of knowledge and a score ranging from 0 to 5 or 0 to 2 defined as a poor level of knowledge on cervical cancer, risk factors, signs, and prevention, respectively. The level of confidence to notice cervical cancer symptoms was measured by asking respondents, “How confident are you that you would notice a cervical cancer symptom?” answers being “not at all confident, not very confident and very confident.”

### Statistical Analysis

We used STATA version 15.0 for data cleaning and analysis. Descriptive statistics were summarized using frequencies and percentages for categorical variables while means and standard deviations for continuous variables, particularly respondent age in years. The log-binomial regressions model was used to estimate the prevalence ratio with 95% confidence intervals (CIs) for factors associated with willingness to cervical cancer self-sample collection. To assess the correlation between independent variables, we used pairwise correlation statistics. A correlation coefficient, r, of more than 50%, would result in removing one of the variables from the adjusted regression analysis. Using stepwise regression, we developed several adjusted analysis models to determine the factors independently associated with a higher prevalence of willingness to cervical cancer self-sample collection. To test nested models, we used the likelihood ratio test. A p-value of less than 5% was considered statistically significant.

## Results

### Characteristics of the study participants

A total of 706 women participated in this study. More than half (53.5%) were from Meru District Hospital, the rest from the Usa River Health Center. The mean age (SD) of respondents was 30.2 (8.0) years. The majority (74.5%) were married/ cohabiting with their partners. Sixty percent had primary education level, and 36.4% were self-employed. Only 20% of all respondents had health insurance. The majority (71%) had between 1–3 pregnancies (Table 1). Women in both centers (Usa River health center and Meru district hospital) were similar in terms of characteristics such as marital status, occupation, having a health insurance and parity.

**Table 1:** Willingness to HPV self-sample collection by respondent characteristics (N=706)

Variables	Overall (n=706)	Willing (n=492)	Not willing (n=214)	p-value*
**Facility**				<0.001
Usa river health center	378 (53.5)	358 (94.7)	20 (5.3)	
Meru District Hospital	328 (46.5)	134 (40.8)	194 (59.2)	
**Age (years)**				<0.001
≤24	190 (26.9)	104 (54.7)	86 (45.3)	
25–34	310 (43.9)	226 (72.9)	84 (27.1)	
≥35	206 (29.2)	162 (78.6)	44 (21.4)	
**Marital status**				0.013
Single	134 (19.0)	80 (59.7)	54 (40.3)	
Married/cohabiting	526 (74.5)	376 (71.5)	150 (28.5)	
Widowed/divorced/separated	46 (6.5)	36 (78.3)	10 (21.7)	
**Education level**				0.009
Primary	424 (60.1)	287 (67.7)	137 (32.3)	
Secondary	221 (31.3)	152 (68.8)	69 (31.2)	
Higher than secondary	61 (8.6)	53 (86.9)	8 (13.1)	
**Occupation**				<0.001
Formal employment	83 (11.8)	71 (85.5)	12 (14.5)	
Self-employed	257 (36.4)	188 (73.2)	69 (26.8)	
Peasant	147 (20.8)	103 (70.1)	44 (29.9)	
No Occupation	219 (31.0)	130 (59.4)	89 (40.6)	
**Has health insurance**	140 (19.8)			0.001
No	566 (80.2)	378 (66.8)	188 (33.2)	
Yes	140 (19.8)	114 (81.4)	26 (18.6)	
**Parity**				0.041
None	77 (10.9)	50 (64.9)	27 (35.1)	
1–3	498 (70.5)	339 (68.1)	159 (31.9)	
More than 4	131 (18.7)	103 (78.6)	28 (21.4)	

* P-value from the Chi-square distribution.

Of the 706 women in the study, over two-thirds (69.7%) stated a willingness to HPV self-sample collect. However, women attending the Usa River Health Center were more unwilling to self-collect than women attending the Meru District hospital (5.3% vs 59.2%). The prevalence of willingness to HPV self-sample collect was statistically significant by, respondent's age (years), marital status, education level, occupation, possession of health insurance, and parity (p<0.05) (Table 1).

### Willingness to HPV self-sample collection by knowledge-related characteristics

The overall prevalence of good knowledge on cervical cancer risk factors, warning signs, and prevention was 37.2%, 22.8%, and 53.4%, respectively. More than half (57%) of the respondents were not very confident to notice cervical cancer symptoms. The prevalence of willingness to HPV self-sampling for cervical cancer screening was significant by respondent knowledge on the risk factors, warning signs, and prevention of cervical cancer, as well as by their level of confidence to notice cervical cancer symptoms (p<0.001) (Table 2).

**Table 2:** Willingness to cervical cancer self-sample collection by knowledge-related characteristics (N=706)

Knowledge related factors	Overall (n=706)	Willing (n=492)	Not willing (n=214)	p-value*
**Knowledge of cervical cancer risk** **factors**				<0.001
Poor	443 (62.8)	286 (64.6)	157 (35.4)	
Good	263 (37.2)	206 (78.3)	57 (21.7)	
**Knowledge of cervical cancer warning** **signs**				<0.001
Poor	545 (77.2)	361 (66.2)	184 (33.8)	
Good	161 (22.8)	131 (81.4)	30 (18.6)	
**Knowledge of cervical** **cancer prevention methods**				<0.001
Poor	329 (46.6)	207 (62.9)	122 (37.1)	
Good	377 (53.4)	285 (75.6)	92 (24.4)	
**Confidence to notice cervical cancer** **symptoms**				<0.001
Not at all confident	208 (29.5)	73 (35.10)	135 (64.90)	
Not very confident	403 (57.1)	337 (83.62)	66 (16.38)	
Very confident	95 (13.5)	82 (86.32)	13 (13.68)	

* P-value from the Chi-square distribution.

### Factors associated with willingness to HPV self-sampling

In the crude (or unadjusted) analysis, high prevalence of willingness to HPV self-collect was observed among respondent from Meru District Hospital (PR=2.32, 95%CI 2.03–2.65) compared to Usa River health center, those who were married/ cohabiting and widowed/ divorced/separated (PR=1.20, 95%CI 1.03–1.39 and PR=1.31, 95%CI 1.07–1.61), compared to single women, respectively. Women aged between 25–34 (PR=1.33, 95%CI 1.15, 1.54) and ≥35 years (PR=1.44, 95%CI 1.24, 1.67) were more willing to HPV self-sample collection compared to younger (≤24 years) women. Those with higher education levels (PR=1.28, 95%CI 1.14–1.44), compared to primary education level, and who reported having health insurance (PR=1.22, 95%CI 1.11–1.35), were also more willing to self-collect. Compared to those with no occupation, self-employed respondents (PR=0.82, 95%CI 0.71–0.94) and peasants (PR=0.69, 95%CI 0.60–0.80) were less likely to be willing to self-collect. Furthermore, a higher prevalence of willingness to self-collect was observed among respondents with good knowledge of cervical cancer risk factors (PR=1.21, 95%CI 1.11–1.33), warning signs (PR=1.23, 95%CI 1.12–1.35), and prevention (PR=1.20, 95%CI 1.09–1.33). Also, those who were not very confident (PR=2.38, 1.97–2.88), and very confident (PR=2.46, 95%CI 2.01–3.01) to notice cervical cancer symptoms were more willing to self-collect (Table 3).

**Table 3:** Factors associated with willingness to HPV self-sample collection (N=706)

Variables	CPR* (95% CI)	p-value	APR^†^ (95% CI)	p-value
**Facility**				
Usa River health center	Ref		Ref	
Meru District Hospital	2.32 (2.03–2.65)	<0.001	2.02 (1.77–2.31)	<0.001
**Age (years)**				
≤24	Ref		∼	
25–34	1.33 (1.15–1.54)	<0.001		
≥35	1.44 (1.24–1.67)	<0.001		
**Marital status**			∼	
Single	Ref			
Married/cohabiting	1.20 (1.03–1.39)	0.018		
Widowed/ divorced/ separated	1.31 (1.07–1.61)	0.010		
**Education level**			∼	
Primary	Ref			
Secondary	1.02 (0.91–1.14)	0.777		
Higher than secondary	1.28 (1.14–1.44)	<0.001		
**Occupation**				
No Occupation	Ref		Ref	
Formal employment	0.86 (0.76–0.96)	0.008	1.22 (1.07–1.37)	0.002
Self-employed	0.82 (0.71–0.94)	0.005	1.09 (0.98–1.20)	0.106
Peasant	0.69 (0.60–0.80)	<0.001	1.21 (1.07–1.36)	0.002
**Has health insurance**			∼	
No	Ref			
Yes	1.22 (1.11–1.35)	<0.001		
**Parity**			∼	
None	Ref			
1–3	1.05 (0.88–1.25)	0.597		
More than 4	1.21 (1.01–1.46)	0.045		
**Knowledge of cervical cancer risk** **factors**				
Poor	Ref		∼	
Good	1.21 (1.11–1.33)	<0.001		
**Knowledge of cervical cancer** **warning signs**				
Poor	Ref		Ref	
Good	1.23 (1.12–1.35)	<0.001	1.11 (1.01–1.22)	0.032
**Knowledge of cervical** **cancer prevention methods**				
Poor	Ref		Ref	
Good	1.20 (1.09–1.33)	<0.001	1.13 (1.04–1.20)	0.006
**Confidence to notice cervical cancer** **symptoms**				
Not at all confident	Ref		Ref	
Not very confident	2.38 (1.97–2.88)	<0.001	1.61 (1.33–1.93)	<0.001
Very confident	2.46 (2.01–3.01)	<0.001	1.36 (1.11–1.67)	0.004

* CPR, Crude Prevalence Ratio. APR, Adjusted Prevalence Ratio: adjusted for the health facility, age, occupation, knowledge on the risk factors, warning signs, and prevention of cervical cancer, and confidence to notice cervical cancer symptoms.

Using the pairwise correlation matrix, we assessed for the correlation between independent variables to enter in the adjusted regression models—none of the variables correlated by even 40%. Hence, we considered all independent variables in the regression models. We used stepwise regression to develop several models adjusted for other factors to determine the independent factors associated with willingness to self-collect. In the first step, we included all covariates (as shown in Table 3 below) in the model, resulting in the dropping of marital status, education level, ownership of health insurance, and parity. Then, we removed the age of women and the good knowledge of cervical cancer risk factors as they did not have any additional effect on the model.

Factors that remained to be significantly associated with a higher prevalence of willingness to cervical cancer self-collection were type of health facility, attending Meru District Hospital compared to Usa River health center (PR=2.02, 95%CI 1.77–2.31), good knowledge about the warnings signs (PR=1.11, 95%CI 1.01–1.22), and prevention of cervical cancer (PR=1.13, 95%CI 1.04–1.20). Not being very confident (PR=1.61, 95%CI 1.33–1.93), and very confident (PR=1.36, 95%CI 1.11–1.67) to notice cervical cancer symptoms. Some sociodemographic factors were also associated with willing to self-collect including formal employment (PR=1.22, 95%CI 1.07–1.37) and peasants (PR=1.21, 95%CI 1.07–1.36) (Table 3).

## Discussion

Findings from a systematic review and meta-analysis show that HPV self-sample collection substantially improves the participation of women who do not routinely attend cervical cancer screening programs^14^. Furthermore, the use of HPV self-sample collection has the potential to address many barriers to screening that women may have^14^. Almost 70% of the women in this study reported a willingness to self-collect. Willingness was associated with type of health facility, good knowledge about cervical cancer, confidence to notice cervical cancer symptoms, having a formal employment, and being a peasant. Overall, the offer to self-collect was well-received, which is likely to increase compliance and the uptake of cervical cancer screening services. These findings are also consistent with other studies where the uptake of cervical cancer screening increased following the application of HPV sample collection procedures for women who do not attend screening programs or services for different reasons^11^, ^15^, ^17^–^19^, ^22^. Women in Kenya, however, preferred clinician-collection over self-collection of HPV samples for cervical cancer screening^16^, probably due to concerns about performing self-collection properly^16^–^18^. In this study, however, we did not assess the feasibility of HPV self-collection and associated challenges.

The type of the health facility was an important predictor for willingness to self-sample collection for cervical cancer screening. We found that women from Meru District hospital were more willing to participate in self-sample collection compared to those from the Usa River Health Center. The Meru District Hospital has been the main cervical cancer screening clinic in Arusha region. Therefore, the surrounding community has been exposed to cervical cancer screening and this could have had an impact on willingness to HPV self-sample collection. Despite the observed associations, interventions to address barriers to self-sample collection such as knowledge of cervical cancer need to be emphasized to promote uptake of screening services^6^, ^10^, ^20^, ^21^. In the present study, women with good knowledge about the signs and prevention of cervical cancer were more willing to accept the HPV self-collect. While knowledge-based interventions on cervical cancer are crucial to increase the uptake of screening services^20^, especially among high-risk women, structural and individual-level barriers, particularly those related to cost of care and access to information also influence uptake^20^.

Furthermore, compared to women with no occupation, those with formal employment as well as peasants were more willing to HPV self-collect. These women might have preferred this testing approach due to the nature of their jobs, i.e., job commitments and engagement in small scale agricultural activities rendering it difficult for them to visit health facilities to access screening programs. Peasant are hard to reach populations and, therefore, this HPV self-collection for them is better than going to the clinic where they have to spend their money on the fare and time.

HPV self-sample collection reduce the number of clients who would go for VIA because only the positive would go for VIA. Women who test positive would need to have more definitive testing done, but this number would be much less than that if we conducted a population-based screening program. HPV self-sample collection, therefore, is an excellent way to focus on those at high-risk of cervical cancer and not inconvenience those who are at low-risk.

This study had several limitations. Firstly, selection bias has to be considered. Participation was voluntary and assessment of willingness to self-collect was self-reported, which may have led to an overestimation of women's favorable opinions of self-sample collection. This study was hospital-based and recruited women who attended outpatient clinics of selected facilities; hence the findings may not be generalized to the general population. The research did not also dwell on factors which would identify barriers towards HPV self-sample collection. The fact that the study included women aged less than 30 years of age might have under- or over-estimated the proportion of willingness to self-collect HPV samples, because the recommended cervical cancer screening age in Tanzania is 30 years and above^25^.

## Conclusion

The majority of women were willing to self-collect HPV samples for cervical cancer screening. Our findings suggest that self-collection is an acceptable and viable means of screening for cervical cancer, which has great implications for Tanzania from a health policy perspective.
